# Effects of a 3-Week In-Hospital Multidisciplinary Body Weight Reduction Program in Obese Females: Is Measured Resting Energy Expenditure Essential for Tailoring Adequately the Amount of Energy Intake?

**DOI:** 10.3389/fnut.2021.678788

**Published:** 2021-05-12

**Authors:** Sofia Tamini, Sabrina Cicolini, Diana Caroli, Alessandro Sartorio

**Affiliations:** ^1^Istituto Auxologico Italiano, Experimental Laboratory for Auxo-Endocrinological Research, Verbania and Milan, Italy; ^2^Istituto Auxologico Italiano, Division of Auxology and Metabolic Diseases, Verbania, Italy

**Keywords:** metabolic rehabilitation, body weight reduction program, resting energy expenditure, indirect calorimetry, diet, obesity

## Abstract

In the obese population, the prescription of a proper diet plan is essential to ensure an appropriate and gradual weight loss, reduce the risk of weight cycling and favor an overall improvement of health conditions. Energy needs are commonly estimated using predictive equations, even if their accuracy is still debated, especially in severely obese subjects. In the present study, 850 severely obese females admitted to our hospital for a multidisciplinary body weight reduction program (BWRP) were divided into three subgroups, “hypo-,” “normo-,” and “hyper-metabolic,” based on the comparison between estimated resting energy expenditure (eREE, using the Mifflin equation) and measured REE (mREE, using indirect calorimetry). The majority of this study population was considered normo-metabolic (59.4%, mREE between 90 and 110% of eREE), 32.6% was hyper-metabolic (mREE > 110% of eREE) and only 8% was hypo-metabolic (mREE < 90% of eREE). The three subgroups were evaluated before and after a 3-week BWRP, entailing energy restricted diet, adapted physical activity, psychological counseling and nutritional education. Since the diet plan during the BWRP consisted of a 30% reduction of total energy expenditure (obtained by multiplying mREE by the physical activity level), each subgroup responded positively to the BWRP independently from the difference between mREE and eREE, the extent of BMI reduction and clinical, metabolic and physical amelioration being comparable among the three subgroups. By contrast, the restriction of the energy intake based on eREE during the BWRP would have determined a slighter caloric restriction in the hypo-metabolic subgroup, thus determining a smaller body weight reduction, and, by contrast, a more marked caloric restriction in the hyper-metabolic subgroup, probably difficult to be tolerated and maintained for prolonged period. In conclusion, the percentage of subjects with “slow metabolism” in a Caucasian female obese population seeking hospitalization for a BWRP is actually lower than expected, finding controverting the common notion that obesity is mostly due to reduced REE. The high percentage (40%) of inadequate eREE in these female obese populations further underlines the absolute need to include the measurement of REE in the clinical practice for the correct prescription of energy intake in severely obese populations.

## Introduction

Obesity prevalence has increased in pandemic dimensions over the past decades, becoming a major health concern worldwide (1). This condition is related with a reduced life expectancy, up to 10 years, and with increased risk of several relevant complications (2), such as type 2 diabetes mellitus, cardiovascular, gastrointestinal and non-alcoholic fatty liver diseases, metabolic syndrome, and different types of cancer (1).

Obesity is characterized by an excessive fat storage, caused by the interaction between genetic, environmental and psychological factors, all together leading to an imbalance between energy intake and energy expenditure producing a positive energy balance (3).

The treatment of obesity requires lifestyle intervention, behavioral therapy and energy deficit achieved throughout caloric restriction and physical exercise (4) in order to reduce body weight and correct wrong habits. In this context, residential body weight reduction programs (BWRP) have been demonstrated to be an effective strategy to counteract this condition and its associated comorbidities, due to their multidisciplinary approach (5, 6). During BWRP, the prescription of a correct diet plan is essential to ensure a proper and gradual weight loss and to determine improvements of body composition and metabolic parameters (5–8).

Many clinical guidelines recommend individually tailored weight loss programs (9–11) that requires an evaluation of individual energy needs in order to create the correct energy deficit. For these reasons, daily total energy expenditure (TEE) assessment is crucial, since it allows to quantify the precise amount of energy necessary for each individual and, consequently, to adopt the correct diet plan (12).

Daily TEE is composed of three main components: (i) resting energy expenditure (REE), which is the major component, especially in sedentary people (i.e., the energy required to support basic metabolic activities) (3); (ii) activity-related energy expenditure, which is strictly dependent on the subjects' lifestyle and includes both voluntary and involuntary physical activity, thus being the most variable TEE component; (iii) diet-induced thermogenesis (13).

In obese patients, which are mostly sedentary, REE determination is essential since it allows to accurately quantify the energy necessary to optimize weight loss and its maintenance (12).

Although indirect calorimetry (IC) is considered the gold standard to measure REE (14), however it has some limitations, being expensive, time-consuming and not always available since it requires expert technician and specific instruments. For this reason, IC is not always used to establish REE, especially in standard clinical contexts. Consequently, as an alternative approach to IC, several predictive equations have been validated both in normal (15), overweight (11) and obese population (12, 16), aiming to estimate REE, based on sex, age, weight, height, and/or body composition. Predictive equations, however, are not always accurate and their use is a matter of debate, especially in obese subjects (17) since their accuracy tends to decrease with increasing body mass index (BMI) (16). To date, the Mifflin equation (18) appears to be the best predictive equation in term of accuracy and bias in obese subjects (11, 12, 16, 17).

In the present study, a large cohort of severely obese females was evaluated before and after a 3-week multidisciplinary BWRP in order to compare the estimate REE (using the Mifflin equation, eREE) with the measured REE (using IC, mREE). The aims of the study were to identify the prevalence of “hypo-,” “normo-,” and “hypermetabolic” obese subjects, throughout the comparison of eREE with mREE, and to evaluate the 3-week BWRP effects on several metabolic and functional parameters in the three subgroups.

## Materials and Methods

### Study Population

A total of 898 females hospitalized at the Division of Metabolic Diseases, Istituto Auxologico Italiano, IRCCS, Piancavallo (VB) for a 3-week multidisciplinary BWRP were eligible for participating in the present study. The inclusion criteria were: females older than 18 years and BMI > 35 kg/m^2^. The exclusion criteria were severe physical inability and/or cognitive impairments hampering the execution of the tests included in the present study.

Because of declining to participate (expressed by the patients), medical reasons and a refusal to complete the 3-week hospitalization period (self-discharge for personal reasons), 48 women were excluded. Taking into account these drop-outs, a total of 850 women were recruited.

The sample size was considered adequate, taking into account a power analysis in which a mean value of ΔBMI (%) after BWRP was supposed to be equal to 4.0 ± 4.0% with an α error of 0.05 at two tails and a power of 0.80.

The study was approved by the Ethical Committee of Istituto Auxologico Italiano, Milan, Italy (Research Code: 01C621, acronym: BEEOB). The purpose and objective of the study were explained to each subject and written informed consent was obtained before the beginning of the study.

### BWRP

All subjects had a full medical history and physical examination.

During the 3-week BWRP, a Mediterranean personalized diet was prescribed in all cases, with an energy content obtained by subtracting ~30% from TEE, which is obtained by multiplying the mREE by the physical activity level during the BWRP. Diet composition was: 18–20% proteins, 50–55% carbohydrates (<15% simple sugar), 27–30% lipids (<8% saturated fat), and ~30 g of fibers. Foods to which the patient declared to be allergic were removed from the menu. Five daily portions of fruits and vegetables were mandatory and a fluid intake of at least 1.5 L/day was recommended. During the BWRP the patients had educational lessons on nutritional aspects, consisting of lectures, demonstrations and group discussions with and without a supervisor, which took place every day throughout the whole BWRP period.

The physical activity program consisted of 5 days per week training including (i) 1-h dynamic aerobic standing and floor exercises with arms and legs, at moderate intensity and under the guidance of a therapist; (ii) either 20–30 min cycloergometer exercise at 60 W, or 3–4 km outdoor walking on flat terrain, according to individual capabilities and clinical status. In addition, subjects had 1 h/day of aerobic free activities at the institution on Saturday and Sunday.

Each patient had psychological sessions led by clinical psychologist 2–3 times per week, which were based on cognitive-behavioral strategies with individual or group sessions.

### REE Measurement and REE Estimation

At the beginning of the BWRP, mREE was measured between 8.00 and 10.00 a.m., in thermo-neutral conditions (room temperature: 22–25°C) using an open-circuit indirect computerized calorimeter equipped with a canopy (Vmax 29, Sensor Medics, Yorba Linda, CA), periodically undergone to quality control tests in order to ensure the reliability of the measurements. The gas analyzers were calibrated before each test using a reference gas mixture made of 15% O_2_ and 5% CO_2_. The subjects were in a fasting state from at least 8 h, were not smoking for at least 1 h and waited 30 min in a sitting position before undergoing REE measurement. mREE was assessed in the supine position for at least 30 min, including an acclimation period of 10 min. The data relative to the acclimation period were discarded. The steady state was defined as at least 5 min <5% variation in respiratory quotient, <10% variation in O_2_ and <10% variation in minute ventilation (19). After the steady state was reached, O_2_ consumption and CO_2_ production were recorded at intervals of 1 min for at least 20 min and averaged over the whole measurement period. REE was calculated from O_2_ consumption and CO_2_ production using the Weir's equation (20).

eREE was estimated using the Mifflin equation (18) as follows:

$$\begin{array}{l} eREE=9.99*weight\;\left(kg\right)+6.25*height\;\left(cm\right)-4.92*age\;\\ \left(years\right)+166*sex-161 \end{array}$$

where sex values are = 1 for males and = 0 for females.

### Anthropometry

Body weight (BW) and height were measured following international guidelines (21) using a scale with a stadiometer (Wunder Sa.Bi., WU150, Trezzo sull'Adda, Italy). BMI was calculated as weight (kg)/height (m)^2^. BW and BMI were determined at the beginning (T1) and at the end (T21) of the BWRP.

Body composition was measured at the beginning of the BWRP using a multifrequency tetrapolar impedance meter (BIA, Human-IM Scan, DS-Medigroup, Milan, Italy) with a delivered current of 800 μA at a frequency of 50 kHz. In order to reduce errors of measurement, special care was paid to the standardization of the variables known to affect measurement validity, reproducibility, and precision. Measurements were performed according to the method of Lukaski (22) (after 20 min resting in a supine position with arms and legs relaxed and not in contact with other body parts) and in strictly controlled conditions.

Waist circumference (WC) was measured at T1 at the midpoint between the last rib and the iliac crest using a flexible tape measure.

### Laboratory and Clinical Measurements

Blood samples were collected after an overnight fast in standard tubes for serum. Glucose, total cholesterol (T-C) and HDL cholesterol (HDL-C) were measured by the same internal laboratory using standard methods.

Systolic (SBP) and diastolic blood pressure (DBP) were measured twice (3-min interval in-between) on the dominant arm with an aneroid sphygmomanometer (TemaCertus, Milan, Italy), using appropriated sized cuffs for obese subjects. The mean values were calculated and rounded to the nearest 5 mmHg value.

All these parameters were determined at T1 and at T21.

### Fatigue Severity Scale

Fatigue severity scale (FSS) is one of the most commonly used self-report questionnaire for the fatigue assessment in chronic diseases, validate by our group in the Italian obese population (23).

FSS consists of 9 items describing the negative effects of fatigue on motivation, exercise, physical functioning, ability to carry out duties, work, family or social life. Responders are asked to rate each statement considering the previous week, using a Likert scale ranging from 1 (strong disagreement) up to 7 (strong agreement). The total score is calculated by adding together the raw score of each item (range 9–63).

Each patient filled the FSS at T1 and T21.

### Stair Climbing Test

The stair climbing test (SCT) is a standardized procedure, previously validated by our group (24–26), used to assess motor control and muscle power. In this test, subjects were asked to climb up ordinary stairs (13 steps of 15.3 cm each, vertical distance 1.99 m) at their highest speed, in accordance to their capabilities. The SCT time (T_SCT_) taken to climb the stairs, one step at a time, starting when the foot was elevated and finishing with the contact of the foot on the last step, was measured with a digital stopwatch. The SCT power (P_SCT_) was calculated by using the following equation (27):

$$\begin{array}{l} P_{SCT}=\frac{weight\;\left(kg\right)*9.81m/s^{2}*h}{T_{SCT}} \end{array}$$

where “h” is height (in m) of the total vertical distance of the stairs.

The SCT was performed at T1 and T21.

### Evaluation of Coronary Heart Disease Risk

The coronary heart disease risk (CHD-R) scores were estimated using a simple coronary disease prediction model developed by Wilson et al. (28), which takes into account sex, age, diabetes, smoking, SBP and DBP, T-C, and HDL-C. CHD-R was assessed at T1 and T21. Blood pressure, T-C, HDL-C levels were considered without regard to the use of antihypertensive or lipid-lowering medications. Diabetes diagnosis was defined if the patient was under treatment with insulin or oral hypoglycemic agents, or if fasting blood glucose levels exceeded 140 mg/dL at T1. Subjects who smoked at least one cigarette per day during the previous 12 months were classified as smokers.

These variables have been demonstrated to be independent and biologically important risk factors for CHD. Additionally, this score is a simple approach to predict risk for initial CHD events in disease-free outpatients at 10 years.

### Statistical Analysis

Continuous variables were expressed as means ± standard deviation. The Shapiro-Wilk test showed that all parameters were normally distributed. Each parameter was evaluated as absolute value and also as percentage of pre-, post-BWRP (i.e., T1 and T21) differences (Δ %), except for CHD-R which was considered in its corresponding unit of measurements (Δ points).

The study population was divided into three subgroups based on the percentage of ratio between mREE and eREE (predicted by the Mifflin equation), as described below. Moreover, each subgroup was further divided into two different age groups to analyze possible age-related differences.

All parameters were compared among these three subgroups, between age groups and before and after BWRP by using a *t*-Student-test for paired data (for pre-, post-comparison) or unpaired data (comparison between age groups) or one-way ANOVA (comparison between subgroups and Δ comparison) followed by *post-hoc* Turkey's test. A level of significance of *p* < 0.05 was used for all data analysis.

## Results

The whole study group (no: 850, age: 49.0 ± 15.0 years, BMI: 43.3 ± 6.0 kg/m^2^) was divided into three subgroups taking into account the relationship between estimated and measured REE: (i) hypo-metabolic: mREE <90% of eREE; (ii) normo-metabolic: mREE between 90 and 110% of eREE; (iii) hyper-metabolic mREE > 110% of eREE.

The majority of the study population was defined as normo-metabolic (59.4%), 32.6% was hyper-metabolic and only 8% was hypo-metabolic.

### Main Characteristics of the Three Subgroups

The main characteristics of the three subgroups are shown in Table 1.

**Table 1:** Characteristic of the three subgroups.

	**Hypo-metabolic**	**Normo-metabolic**	**Hyper-metabolic**
	**(<90%)**	**(90–110%)**	**(>110%)**
No.	68 (8%)	505 (59.4%)	277 (32.6%)
Age (yrs)	46.2 ± 15.1	48.3 ± 15.4	51.1 ± 14.2^*^
mREE (kcal/day)	1480.0 ± 206.0	1692.6 ± 243.2	1915.3 ± 274.1
eREE Mifflin (kcal/day)	1738.8 ± 202.9	1684.1 ± 231.5	1611.2 ± 213.9
WC (cm)	121.5 ± 12.8	120.3 ± 13.0	119.2 ± 11.9
BW (kg)	113.2 ± 17.1	109.5 ± 104.7	104.5 ± 15.8^**^
BMI (kg/m^2^)	44.6 ± 6.7	43.7 ± 6.1	42.5 ± 5.5^*^
FM (kg)	60.9 ± 15.3	56.4 ± 14.1	52.0 ± 12.8^**^
FFM (%)	47.0 ± 5.5	48.8 ± 5.5	50.6 ± 5.9^**^

*mREE, measured Resting Energy Expenditure; eREE, estimated Resting Energy Expenditure; WC, Waist Circumference; BW, Body Weight; BMI, Body Mass Index; FM, Fat Mass; FFM, Fat-Free Mass*.

* *p < 0.05 compared to the other subgroups*.

** *p < 0.01 compared to the other subgroups*.

The hyper-metabolic subgroup was significantly older and lighter (lower BW and BMI) as compared to the other two subgroups (*p* < 0.05), while the normo-metabolic and the hypo-metabolic groups were comparable in term of age, BW, and BMI. The analysis of the body composition showed that the hyper-metabolic subgroup had the best body composition profile, with a greater amount of fat-free mass (FFM, *p* < 0.01) and lower fat mass (FM, *p* < 0.01). All three subgroups were comparable in term of WC.

As expected, no significant difference was found comparing mREE with eREE in the normo-metabolic subgroup, while the hypo-metabolic subgroup showed a significantly lower mREE (approx. −260 kcal, *p* < 0.001) and the hyper-metabolic one a significantly higher mREE (approx. +300 kcal *p* < 0.001) as compared to the corresponding eREE.

### Subdivision into Age groups

The three subgroups were further divided into two groups according to age: females aged from 20 to 49 years and females aged from 50 to 80 years. The characteristics of these two age-dependent subgroups are reported on Table 2.

**Table 2:** Characteristics of the three subgroups divided into age groups.

	**Hypo-metabolic**		**Normo-metabolic**		**Hyper-metabolic**	
	**(<90%)**		**(90–110%)**		**(>110%)**	
**Age range**	**20–49**	**50–80**	**20–49**	**50–80**	**20–49**	**50–80**
*n*.	33	35	236	269	109	168
mREE (kcal/day)	1578.8 ± 193.9^*^	1386.9 ± 172.8	1811.5 ± 239.6^*^	1588.25 ± 193.7	2069.4 ± 267.7^*^	1815.4 ± 228.8
eREE Mifflin (kcal/day)	1819.7 ± 193.0^*^	1662.6 ± 183.6	1810.9 ± 215.6^*^	1572.9 ± 182.8	1745.8 ± 195.0^*^	1523.8 ± 177.5
BW (kg)	114.2 ± 16.9	112.3 ± 17.4	113.5 ± 18.3^*^	105.9 ± 15.5	108.8 ± 17.1^*^	101.7 ± 14.3
BMI (kg/m^2^)	44.5 ± 6.2	44.7 ± 7.2	43.7 ± 6.4	43.7 ± 5.9	42.6 ± 6.2	42.5 ± 5.0
mREE/BW (kcal/kg)	13.9 ± 0.8^*^	12.5 ± 1.2	16.1 ± 1.2^*^	15.1 ± 1.1	19.2 ± 1.7^*^	17.9 ± 1.3
mREE/kg FFM (kcal/kg)	30.0 ± 3.1^*^	26.9 ± 2.6	33.8 ± 4.0^*^	30.7 ± 3.0	38.7 ± 4.6^*^	35.5 ± 4.8

*mREE, measured Resting Energy Expenditure; eREE, estimated Resting Energy Expenditure; BW, Body Weight; BMI, Body Mass Index; FFM, Fat-Free Mass*.

* *p < 0.01 compared to the other age subgroup within the same metabolic group*.

In the three subgroups (hypo-normo-hyper-metabolic), younger patients had a significantly higher mREE and eREE (*p* < 0.01). Younger normo-metabolic and hyper-metabolic females had a significantly higher BW (*p* < 0.01), while no age-related differences in BW were found in the hypo-metabolic group. BMI was comparable between younger and older patients in all three subgroups. Moreover, calorie consumption per kilo, both mREE/BW and mREE/FFM was significantly higher in younger patients in all three subgroups (*p* < 0.01).

### Three-Week BWRP Effects in the Three Subgroups

Table 3 shows the metabolic and functional effects of the 3-week BWRP in the three subgroups.

**Table 3:** Effect of the BWRP on the three subgroups.

**Parameter**	**Pre**	**Post**	**Δ %**	***p-*value**
		**Hypo-metabolic (no. 68)**		
BW (kg)	113.2 ± 17.1	108.3 ± 16.2	4.3 ± 1.2	<0.001
BMI (kg/m^2^)	44.6 ± 6.7	42.7 ± 6.4	4.3 ± 1.2	<0.001
Glucose (mg/dl)	88.4 ± 17.4	82.9 ± 20.8	6.2 ± 11.1	<0.01
T-C (mg/dL)	189.2 ± 36.8	167.5 ± 34.9	10.8 ± 11.3	<0.001
HDL-C (mg/dL)	50.5 ± 11.1	44.2 ± 10.3	11.4 ± 14.0	<0.001
SBP (mmHg)	124.6 ± 13.9	117.1 ± 9.3	5.2 ± 8.3	<0.001
DBP (mmHg)	76.4 ± 8.3	72.9 ± 6.8	3.8 ± 11.3	<0.01
FSS score (points)	35.4 ± 11.9	27.8 ± 11.2	21.0 ± 17.5	<0.001
SCT time (sec)	6.3 ± 1.6	5.9 ± 1.5	4.5 ± 6.0	<0.001
SCT power (W)	369.9 ± 95.3	376.0 ± 96.7	0.6 ± 8.4	ns
CHD-R (points)	4.7 ± 6.0	4.5 ± 6.3	0.2 ± 2.7	ns
		**Normo-metabolic (no. 505)**		
BW (kg)	109.5 ± 17.3	104.7 ± 16.4	4.3 ± 1.5	<0.001
BMI (kg/m^2^)	43.7 ± 6.1	41.8 ± 5.9	4.3 ± 1.5	<0.001
Glucose (mg/dl)	90.4 ± 23.0	81.5 ± 15.2	8.1 ± 11.6	<0.001
T-C (mg/dL)	190.7 ± 36.7	167.4 ± 31.8	11.3 ± 12.7	<0.001
HDL-C (mg/dL)	50.3 ± 12.7	42.8 ± 9.9	13.6 ± 12.3	<0.001
SBP (mmHg)	127.1 ± 13.8	120.6 ± 9.1	4.4 ± 9.4	<0.001
DBP (mmHg)	76.7 ± 7.5	74.1 ± 6.4	2.7 ± 11.1	<0.001
FSS score (points)	34.9 ± 12.5	26.8 ± 11.0	21.8 ± 18.8	<0.001
SCT time (sec)	5.9 ± 1.8	5.7 ± 1.8	3.6 ± 4.2	<0.001
SCT power (W)	400.2 ± 135.6	398.1 ± 137.2	0.7 ± 5.2	ns
CHD-R (points)	6.0 ± 6.0	5.8 ± 6.1	0.2 ± 2.4	ns
		**Hyper-metabolic (no. 277)**		
BW (kg)	104.5 ± 15.8	100.1 ± 15.0	4.2 ± 1.5	<0.001
BMI (kg/m^2^)	42.5 ± 5.5	40.8 ± 5.3	4.2 ± 1.5	<0.001
Glucose (mg/dl)	97.3 ± 31.7	85.2 ± 19.6	9.6 ± 14.3	<0.001
T-C (mg/dL)	194.9 ± 35.7	170.3 ± 35.7	12.0 ± 13.8	<0.001
HDL-C (mg/dL)	48.3 ± 11.9	40.9 ± 9.9	14.1 ± 12.7	<0.001
SBP (mmHg)	127.5 ± 13.5	121.1 ± 9.1	4.3 ± 9.3	<0.001
DBP (mmHg)	76.7 ± 7.7	74.3 ± 6.5	2.7 ± 10.8	<0.001
FSS score (points)	36.7 ± 13.1	28.1 ± 11.7	21.9 ± 21.1	<0.001
SCT time (sec)	6.0 ± 1.8	5.8 ± 1.7	4.1 ± 4.4	<0.001
SCT power (W)	366.7 ± 122.0	367.2 ± 126.6	0.2 ± 5.4	ns
CHD-R (points)	7.8 ± 5.7	7.6 ± 5.5	0.1 ± 2.6	ns

*BW, body weight; BMI, body mass index; T-C, total cholesterol; HDL-C, HDL cholesterol; SBP, systolic blood pressure; DBP, diastolic blood pressure; FSS, fatigue severity scale; SCT, stair climbing test; CHD-R, coronary heart disease risk. Δ CHD-R is expressed in points*.

Independently from the difference between mREE and eREE, each subgroup responded positively to the BWRP. In fact, BWRP significantly reduced BW and BMI, glycaemia, T-C, HDL-C, SBP, DBP, FSS and T_SCT_, and in a comparable manner in the three subgroups. By contrast, BWRP did not significantly change P_SCT_ and CHD-R in the three subgroups.

**Figure 1:**
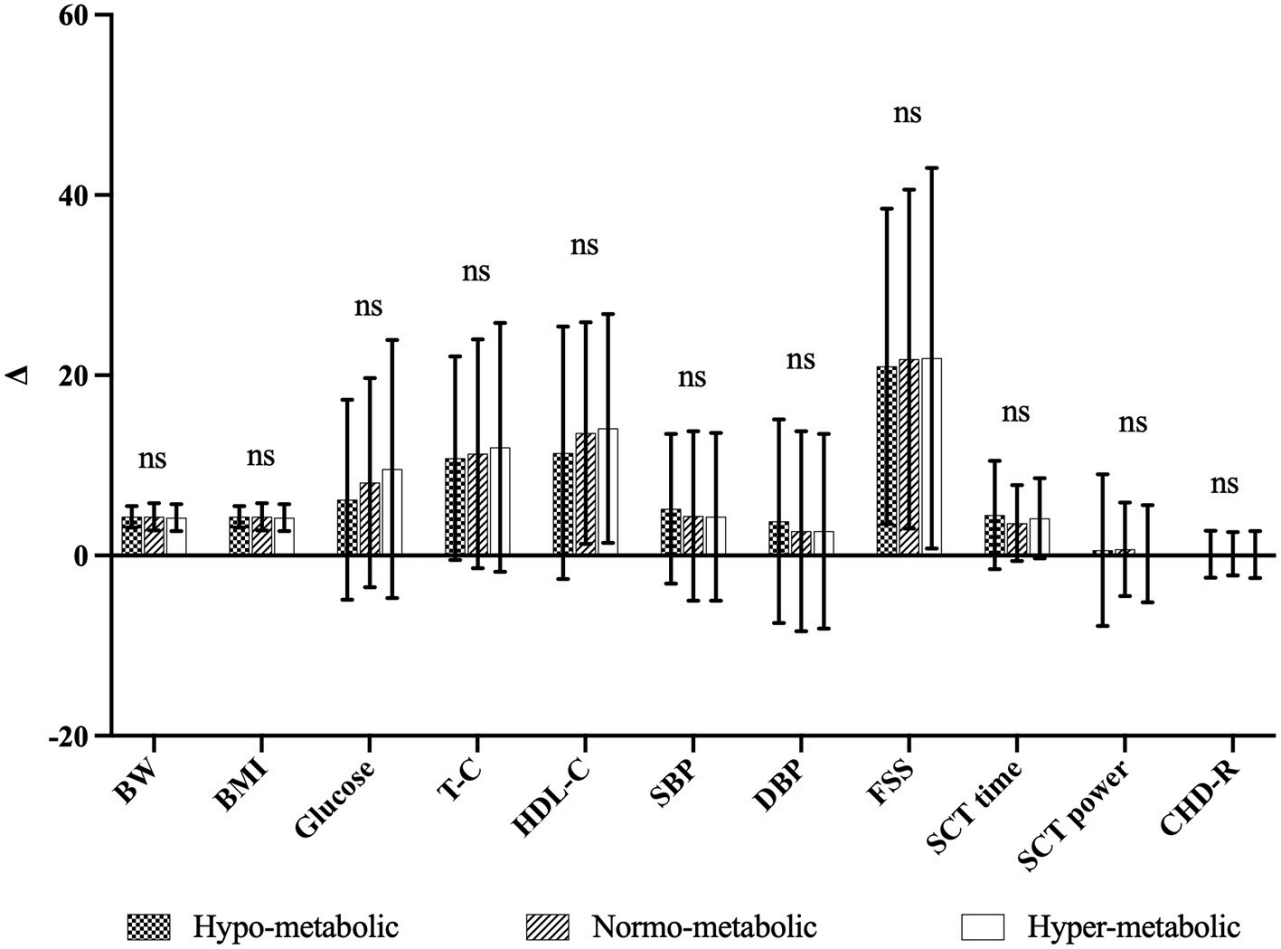
Comparison of Δ-values of the three subgroups. ns, not significant, Δ, differences between pre-, post-BWRP (i.e., T0 and T21). Δ-values are expressed as Δ% except for CHD-R in Δ points.

In addition, as shown in Figure 1, when the Δ % values of each parameter (for CHD-R Δ points) were compared, no significant differences were found among the three subgroups, indicating a similar effectiveness of the BWRP intervention independently from the different mREE.

## Discussion

Obese subjects are thought to have lower energy expenditure compared to normal-weighted individuals, determining the popular notion that obesity is associated to a “slow metabolism” (3). In fact, some longitudinal studies support the idea that reduced energy expenditure is a risk factor for obesity development (29). However, this hypothesis is still debated, since other studies suggested that obese individuals might have higher REE compared to normal-weighted subjects, most likely because of their increased BW (30, 31).

In the present study, the majority of the obese females recruited was normo-metabolic (mREE between 90 and 110% of eREE), more than 30% was hyper-metabolic (mREE > 110% of eREE) while, surprisingly, only 8% of the study population was hypo-metabolic (mREE <90% of eREE). Therefore, these results suggest that low REE and “slow metabolism” are not as implicated as previously believed in causing obesity and in maintaining this condition, considering that many subjects recruited were actually hyper-metabolic and obese anyway. Thus, although obese individuals might spend more energy performing daily life activity, due to their greater weight burden, their TEE is lower as a consequence of a sedentary lifestyle (3).

Interestingly, the hyper-metabolic group was slightly older than the other subgroups, this finding being quite surprising taking into account the fact that REE is reported to decline with age (32, 33). This subgroup was also lighter in term of both BW and BMI, showing also a greater amount of FFM, one of the most important contributor to REE (34). Considering the age-dependent effects in the three subgroups (females aged from 20 to 49 years and from 50 to 80 years), younger patients had the highest absolute REE, this pattern being confirmed also normalizing REE per body mass (i.e., mREE/BW) and FFM (i.e., mREE/FFM) (35). The normalization of REE per unit of BW/FFM further reinforces the finding that younger patients of this specific study population had the highest absolute REE irrespectively from their hypo-, normo-, or hyper-metabolic status.

Although the great efforts to identify valuable and accurate equations to estimate REE in the obese population, the present study further highlights their inappropriateness. In fact, the Mifflin equation, which is considered the most accurate equation for the obese individuals (11, 16, 17), wrongly predicted REE in 40% of the patients recruited, with a mREE either <90% or >110% of eREE. This percentage is awfully high and further stresses the need for a precise REE assessment to ensure correct caloric intakes (14), especially in severely obese population, in order to prevent under- or over-feeding and their related consequences (i.e., too rapid weight loss causing FFM depletion and increased risk of withdrawal or, by contrast poor weight loss or weight maintenance/gain). For these reasons, the calorie restriction should be defined measuring REE through IC, the actual gold standard technique, rather than estimating REE throughout the commonly available equations.

During the 3-week BWRP, the energy content of the diet was defined by subtracting ~30% from TEE, which was obtained by multiplying the measured REE by the physical activity level during the BWRP. Using this tailored nutritional approach, the whole study population responded positively, independently from the difference between mREE and eREE, with a reduction in BW and BMI and an improvement in several metabolic and clinical parameters, such as glycaemia, T-C, SBP, DBP, and physical parameters, such as FSS (self-perceive fatigability) and T_SCT_ (lower limb muscle power). Moreover, the extent of BW and BMI reduction and clinical, metabolic and physical amelioration were comparable among the three subgroups, indicating a similar effectiveness of the BWRP intervention regardless of the relationships between eREE and mREE.

Although the positive short-term effects of BWRP on all these parameters are actually promising, it will be necessary to verify their maintenance (or not) in the long term.

In our study population, the restriction of the energy intake based on the eREE would have determined a slighter caloric restriction in the hypo-metabolic subgroup (a mean deficit of 400 kcal instead of 680 kcal), thus determining a smaller body weight reduction and, by contrast, a more marked caloric restriction in the hyper-metabolic subgroup (a mean deficit of 1,200 kcal instead of 880 kcal), probably difficult to be tolerated and maintained for prolonged period.

Lastly, it is worth noting to underline the strengths of the present study which are undoubtedly the huge study population of severely obese females (850 patients), the quality of the intervention (i.e., in-hospital multidisciplinary BWRP) and the use of indirect calorimetry, the gold standard technique to measure REE. However, we should mention also the limitation related to the short intervention time (3 weeks) which unfortunately cannot provide information regarding the possibility of maintaining the positive results in the long term. As a second limitation, the present study cannot rule out the possible interference on the results of the drugs taken by the patients for chronic use, since this information was not collected in the present database.

In conclusion, in the obese population the percentage of subject with “slow metabolism” (i.e., hypo-metabolic) is actually much lower than expected, a finding especially relevant in the clinical practice where health professionals are frequently confronted with obese individuals who believe that their condition is mostly due to a reduced REE. The under- and over-estimation of REE, even if by using the best predictive equations, might be one of the reasons explaining the high frequency of failure/drop-out of weight loss programs in the obese population. The correct prescription of the daily restricted energy intake can be obtained by measuring REE with indirect calorimetry, which can allow to accurately quantify the real energy needs in order to provide an adequate nutritional intake and, consequently, optimize weight loss and its maintenance.

The high percentage (~40%) of inadequate REE estimations (in comparison with mREE) in this Caucasian female obese population seeking hospitalization for a BWRP underlines further the need to include this evaluation in the correct prescription of energy intake in severely obese population.

The use of mREE to design the personalized nutritional intervention in our 3-week multidisciplinary BWRP allowed to guarantee to all the obese patients, independently from their baseline metabolism (hyper-, normo-, hypo-), positive and comparable multisystemic effects (i.e., an ~4% BW reduction, muscle-skeletal, and cardiometabolic improvements).

All these results, which obviously require to be maintained over the time, are fundamental in the obese population in order to ameliorate their clinical conditions, to counteract metabolic complications and to improve autonomy and quality of life.

## Data Availability Statement

The raw data generated and analyzed in the present study are available from the corresponding author on reasonable request.

## Ethics Statement

The studies involving human participants were reviewed and approved by the Ethical Committee of Istituto Auxologico Italiano, Milan, Italy (Research Code: 01C621, acronym: BEEOB). The patients/participants provided their written informed consent to participate in this study.

## Author Contributions

ST and AS: conceptualization. ST, DC, and SC: data curation. ST: formal analysis and writing—original draft. AS: funding acquisition and project administration. ST, DC, SC, and AS: investigation, writing—review, and editing. All authors: contributed to the article and approved the submitted version.

## Conflict of Interest

The authors declare that the research was conducted in the absence of any commercial or financial relationships that could be construed as a potential conflict of interest.
